# Well-Being and All-Cause Mortality in Aging Women in the Women's Health Initiative (WHI)

**DOI:** 10.1093/geroni/igab046.1820

**Published:** 2021-12-17

**Authors:** Kenneth Pike, Barbara Cochrane, Nancy Woods, Eileen Rillamas-Sun

**Affiliations:** 1 University of Washington, Seattle, Washington, United States; 2 Fred Hutchinson Cancer Research Center, Seattle, Washington, United States

## Abstract

To study the relationship between well-being and all-cause mortality, we estimated mortality among women in four classes of well-being using the well-being profile from the Women’s Health Initiative Study (WHI). Demographic characteristics were self-reported at enrollment (1993-98). All-cause mortality included death from any cause between 2012-2020. We used logistic regression to examine all-cause mortality risk across the classes, using Class 4 (highest hedonic and eudaemonic well-being scores) as the referent, adjusting for age and race. Compared to Class 4, all other classes had higher age- and race-adjusted odds of death. Highest risks were in Class 1 women (OR=2.61; 95% CI: 2.46-2.76) and Class 3 women (OR=1.62; 95% CI: 1.55-1.68). Women in Class 4 had the lowest risk of all-cause mortality over an 18-year follow-up. These results confirm the utility of a profile of well-being for predicting all-cause mortality while preserving ability to identify the differences among well-being indicators across classes.

